# TFPP: An SVM-Based Tool for Recognizing Flagellar Proteins in *Trypanosoma brucei*

**DOI:** 10.1371/journal.pone.0054032

**Published:** 2013-01-17

**Authors:** Xiaobai Zhang, Yuefeng Shen, Guitao Ding, Yi Tian, Zhenping Liu, Bing Li, Yun Wang, Cizhong Jiang

**Affiliations:** 1 Department of Bioinformatics, the School of Life Sciences and Technology, Tongji University, Shanghai, China; Indiana University, United States of America

## Abstract

*Trypanosoma brucei* is a unicellular flagellated eukaryotic parasite that causes African trypanosomiasis in human and domestic animals with devastating health and economic consequences. Recent studies have revealed the important roles of the single flagellum of *T. brucei* in many aspects, especially that the flagellar motility is required for the viability of the bloodstream form *T. brucei*, suggesting that impairment of the flagellar function may provide a promising cure for African sleeping sickness. Knowing the flagellum proteome is crucial to study the molecular mechanism of the flagellar functions. Here we present a novel computational method for identifying flagellar proteins in *T. brucei*, called trypanosome flagellar protein predictor (TFPP). TFPP was developed based on a list of selected discriminating features derived from protein sequences, and could predict flagellar proteins with ∼92% specificity at a ∼84% sensitivity rate. Applied to the whole *T. brucei* proteome, TFPP reveals 811 more flagellar proteins with high confidence, suggesting that the flagellar proteome covers ∼10% of the whole proteome. Comparison of the expression profiles of the whole *T. brucei* proteome at three typical life cycle stages found that ∼45% of the flagellar proteins were significantly changed in expression levels between the three life cycle stages, indicating life cycle stage-specific regulation of flagellar functions in *T. brucei*. Overall, our study demonstrated that TFPP is highly effective in identifying flagellar proteins and could provide opportunities to study the trypanosome flagellar proteome systematically. Furthermore, the web server for TFPP can be freely accessed at http:/wukong.tongji.edu.cn/tfpp.

## Introduction

The flagellated protozoan parasite Trypanosma brucei is a pathogen agent of human African trypanosomiasis, also known as sleeping sickness. Though the parasite has been known for more than a century, the disease control remains poor and the drugs currently used are highly toxic with serious side effects [1], [2]. *T. brucei* has a digenetic life cycle alternating between a tsetse fly and a mammal host, and motility of the extracellular pathogen is pivotal to the life cycle development and disease pathogenesis. In recent years, the single flagellum of *T. brucei* has been demonstrated as an essential and multifunctional organelle with critical roles in motility, host cell attachment, sensory perception, cell morphogenesis, cell division and host-parasite interaction ([3], [4]). In addition, recent studies have revealed that the flagellar motility is required for the viability of both the insect-form and the bloodstream-form *T. brucei* [5], [6], [7], suggesting that flagellar function analysis may uncover potential novel drug targets. Besides some unique features which may be exploited as drug targets, the *T. brucei* flagellum possesses a canonical 9+2 microtubule axoneme which is conserved among the flagellated eukaryotes. Functional analyses of trypanosome flagellar proteins have provided novel insights into flagellum functions as well as human ciliary diseases, indicating that *T. brucei* provides an excellent model system for dissecting flagellum biology in eukaryotes [3], [5], [8].

Though many studies have revealed the multifunctional nature of the trypanosome flagellum as stated above, the underlying molecular mechanisms are still unclear and the component of the flagellar proteome needs to be identified. As we know, flagellar proteins are all nucleus-encoded, initially synthesized in cytoplasm and then transported to the flagellum. In the past decade, a variety of computational methods have been developed for predicting protein subcellular localization [9], [10], [11], [12], [13]. However, most of the existing tools focus on proteins targeted to major locations such as endoplasmic reticulum, mitochondria, nucleus, and so on. These tools do not provide any information on proteins targeted to more specialized organelles like flagellum. To the best of our knowledge, only a few methods provide predictions for flagellar proteins in prokaryotes [14], [15]. Moreover, no similar prediction tools are available for eukaryotic flagellar proteins. Flagellum is a relatively “closed” organelle and can best be compared with the nucleus considering the entry and exit activities [16]. Though the flagellar membranes are contiguous with the plasma membrane, they are functionally distinct membrane domains with distinct composition and biochemical properties [3]. Therefore, there must be specific targeting and importing mechanisms for flagellar proteins, which are still unknown. Recent proteomic studies have revealed a large number of flagellar proteins in trypanosomes, greatly expanding the inventory of known flagellar proteins [5], [17], [18]. However, due to technical limitations for purification of the intact flagellum from *T. brucei*, a lot of flagellar proteins fail to be detected and many detected proteins can not be assigned to flagellum with certainty.

In this study, we developed a computational method TFPP to identify flagellar proteins in *T. brucei* based on sequence-derived features. We collected a set of flagellar and non-flagellar proteins that have been annotated with high confidence, and selected a number of discriminating properties from various sequence and structural features using a feature selection procedure. On the basis of these features, we developed a support vector machine (SVM)-based classifier to predict flagellar proteins in *T. brucei*. Our results indicate that our method performs well in identifying flagellar proteins and would help to uncover the flagellar proteome in *T. brucei*. We compared the expression profiles of the *T. brucei* proteome at three important life cycle stages, and found that the expression of ∼45% of the expressed flagellar proteins changes greatly during life cycle, indicating life cycle stage-specific regulation of flagellar functions in *T. brucei* which is consistent with previous studies [3].

## Materials and Methods

### Data collection

Data used in this study were retrieved from GeneDB [19] by June 2012. To ensure data quality, we took the information of “Curation” and “Gene Ontology” from GeneDB into account, and only selected the proteins with consistent supporting information. Finally, 156 *T. brucei* proteins were collected as flagellar proteins of high quality based on the comprehensive annotation from GeneDB. To generate a negative dataset for the classification, we extracted *T. brucei* proteins containing annotation for ‘cellular component’ from GeneDB together with the mitochondrial proteins collected in our previous study [9]. This set was filtered by removing the entries either annotated as flagellar related or with low confidence such as “by similarity”, “potential” and “probable”. We retained 652 proteins as non-flagellar proteins with high confidence. To obtain a non-redundant dataset, BLASTclust [20] was used to remove redundant proteins with sequence identity higher than 30%, and 8 flagellar and 60 non-flagellar proteins were discarded from the collected dataset. Thus, 148 flagellar and 592 non-flagellar proteins were finally used as our positive and negative sets, respectively. Systematic IDs of these positive and negative samples are listed in Table S1.

We randomly selected 3/4th of the positive and negative data as the training set. The remaining data were used as the test set. To assess the performance and stability of the prediction model, we repeated the random sampling process fifty times, and obtained 50 groups of training and test sets.

### Feature construction

We examined a number of features which are potentially useful for the identification of flagellar proteins based on the general understanding of protein subcellular localization. The initial features can be grouped into five categories: (a) basic sequence attributes such as sequence length, amino acid composition and di-peptide composition; (b) physicochemical and biochemical properties, such as extinction coefficient, instability index, aliphatic index, and various amino acid propensities obtained from AAindex (http://www.genome.ad.jp/aaindex) [21]; (c) structural properties such as secondary structural content [22], unfoldability and disordered regions [23]; (d) signal peptide [24] and transmembrane topology [25], [26]; (e) post-translational modifications such as phosphorylation [27], acetylation [28] and palmitoylation [29]. Amino acid composition reflects the fraction of amino acids in a protein sequence, while di-peptide composition also encapsulates information about the local order of amino acids in a protein sequence. AAindex is a database of numerical indices representing various physicochemical and biochemical properties of amino acids, currently containing 544 amino acid indices derived from published literature. 544 properties were obtained for each protein by calculating the average value of each amino acid index across the whole protein sequence. The details of the initial features and the computer programs used to calculate them are listed in Table S2. Note that some of these features are represented by multiple feature elements. For example, the amino acid composition of a protein sequence is represented by 20 feature elements. In total, 21 features are considered in our initial feature list, which are represented using 1000 feature elements (Table S2).

### Feature selection and classification

Support vector machine (SVM) is a very useful machine learning method, which has been widely used to solve biological problems such as protein-protein interaction prediction [30], protein subcellular localization prediction [9], post-translational modification recognition [31], biomarker identification in cancer research [32], etc. In this study, SVM with the popular non-linear Gaussian Radial Basis Function kernel (RBF) was used to build the classifier for distinguishing flagellar proteins from non-flagellar proteins. The SVM software we used is LIBSVM (http://www.csie.ntu.edu.tw/~cjlin/libsvm/) which is currently one of the most widely used SVM software. A grid search-based method was used to automatically optimize the two parameters 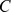 and 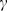 in the training procedure of each SVM classifier, and the search spaces for 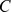 and 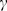 are 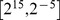 and 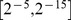 with steps being 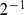 and 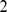, respectively. Codes for parameter selection are publicly available from LIBSVM package.

It is widely appreciated that feature selection in classification is very important not only for reducing running time but also for improving performance and mining useful feature elements which are really relevant to the classification problem. We proposed a feature-selection procedure combining filter and wrapper methods to select a subset of feature elements which can make the classifier achieve best prediction performance. In the first step, F-score was used to measure the discriminative power of each feature element between the positive and negative sets, which is defined as follows,
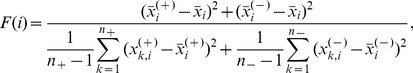
(1)where 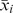, 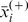 and 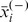 are the average value of the 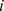
th feature over the whole, positive and negative datasets, respectively; 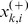 and 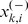 are the 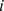
th feature of the 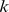
th protein in the positive and negative datasets, respectively; and 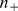 and 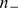 are the numbers of proteins in the positive and negative datasets, respectively. The larger an F-score is, the more discriminative the feature is. In the first round, feature elements with F-scores above a pre-selected threshold were retained and used in the next step feature selection. The F-score threshold was selected based on the distribution of the sorted F-scores of all feature elements, and the cross-validation accuracy of the SVM-based classifier with the retained feature elements should be no worse than that with all of the initial feature elements. The goal of the F-score-based feature selection is to reduce search space by removing a large number of feature elements irrelevant or negligible to our classification problem.

In the second step, we utilized an SVM-based wrapper method using sequential backward selection (SBS) search strategy to find an optimal subset of feature elements that gives the highest cross-validation accuracy of the SVM classifier. Basically, the SBS algorithm starts with the feature set obtained from the F-score-based selection step, and for each iteration, the worst feature element (concerning the cross-validation accuracy of the SVM classifier) is eliminated from the current feature set until only one feature element left. Based on the results of all iterations, the set of feature elements which gives the best performance will be used to build the final classifier model.

### Performance evaluation

Using the selected feature set, SVM-based classifiers were obtained by training on the training sets and were tested on the corresponding test sets. Four common measures were used to evaluate the prediction performance of the trained classifiers, namely sensitivity, specificity, accuracy and the Matthews correlation coefficient (MCC) [33]. MCC is a comprehensive indicator of prediction performance, besides, it can well reflect the balance between the sensitivity and the corresponding specificity. MCC = 1 indicates a perfect prediction, while -1 indicates a completely opposite prediction. Thus, the classifier with the highest MCC was selected as the final prediction model, which is referred to as trypanosome flagellar protein predictor (TFPP for short).

To evaluate the reliability of the predicted result, we analyzed the correlation between the prediction score (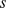) and prediction precision (PP) based on the prediction result of the whole positive and negative sets. Prediction score, that is the decision value obtained from the SVM classifier, reflects the distance between the input vector and the decision plane, thus it is closely related with the prediction reliability. Generally, the higher the absolute decision value is, the more reliable the prediction is. Prediction precision is also known as positive predictive value for positive prediction and negative predictive value for negative prediction respectively, which is calculated as follows:
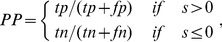
(2)where 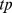 and 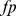 are the numbers of true and false positive samples respectively, while 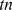 and 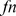 are the numbers of true and false negative samples respectively. We defined three levels of prediction confidence based on prediction precision, namely high with 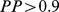, medium with 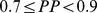 and low with 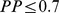.

## Results and Discussion

### Feature contribution

Based on previous studies and our understanding on protein subcellular localization, we collected various types of features that may be relevant to the targeting of flagellar proteins. In total, 21 features represented by 1000 feature elements from all data were taken into account in our initial feature list (Table S2). To ascertain which of the initially considered features are actually effective in discriminating flagellar proteins from non-flagellar proteins, we used an effective feature selection method introduced in the “Materials and Methods” section to remove features irrelevant or negligible to our classification problem. Using this method, a total of 37 feature elements were selected to train the final classifier. Details about these selected feature elements with F-scores and p-values by ANOVA are available in Table S3, and all of these features show significant differences (p-value<10^−5^) between flagellar and non-flagellar proteins. Among these selected features, we found that physicochemical properties play dominant roles in distinguishing flagellar proteins from the other proteins. Flagellar proteins tend to be negatively charged, hydrophilic and thus show higher surface accessibility. Besides, flagellar proteins are rich in the negatively charged residue, glutamic acid. As revealed by an early study, glutamic acid is involved in glutamylation that extensively exists in subpellicular and flagellar microtubules [34].

### Performance of the classifier

**Figure 1:**
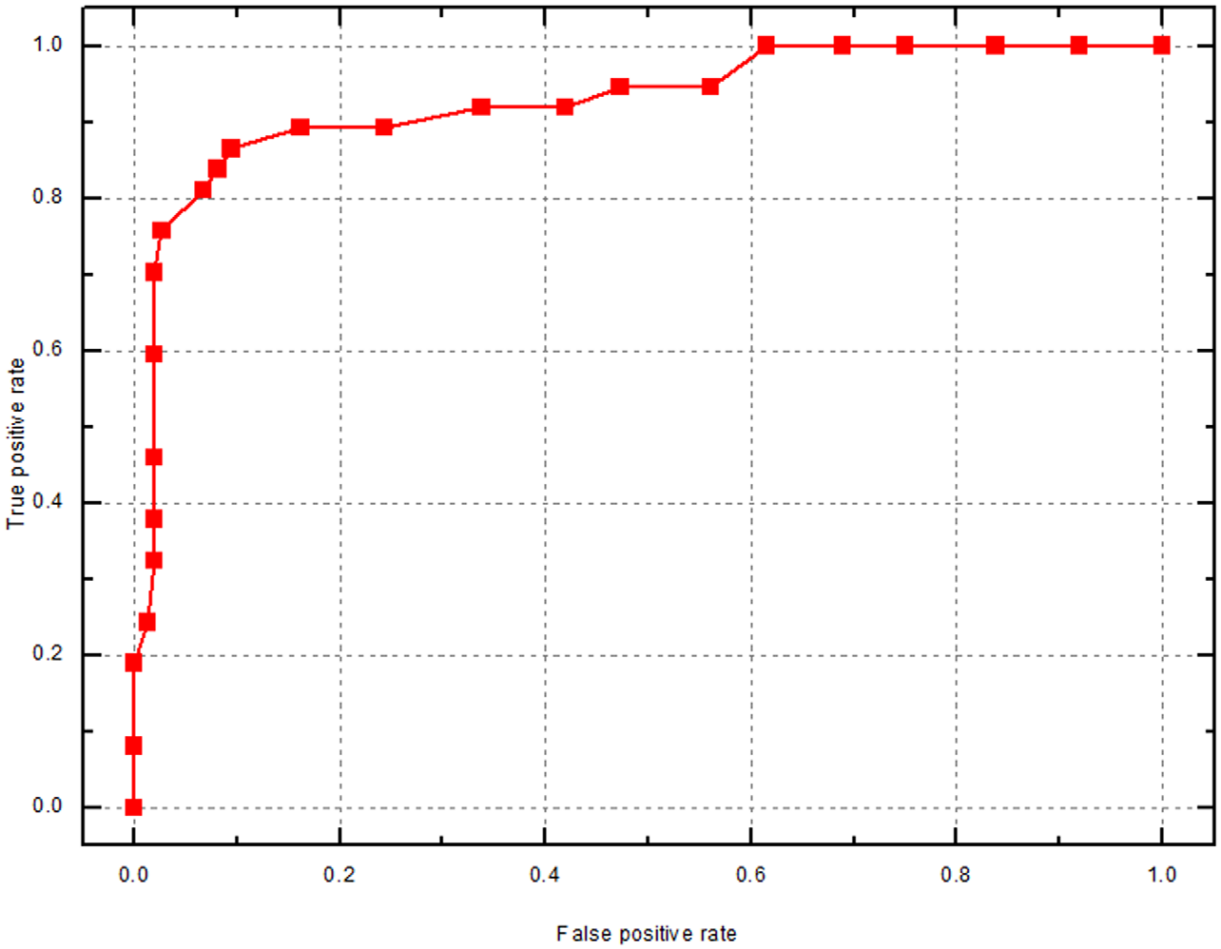
ROC curve of TFPP. AUC is 0.9272.

SVM-based classifiers were built using the 37 selected feature elements which are closely related to the targeting of flagellar proteins. To assess the effectiveness of the selected features as well as the stability of the prediction performance, we trained 50 SVM-based models using the randomly selected training sets and tested these models on the corresponding test sets. As shown in Table 1, the performances of these classifiers are generally consistent with MCC ranging from 0.546 to 0.717. Our final classifier model, TFPP, achieves a total prediction accuracy of 90.3% with sensitivity being 83.8% and specificity being 92.6%. Based on the receiver operating characteristic (ROC) curve, the AUC of TFPP is 0.927, indicating its good performance in recognizing both flagellar and non-flagellar proteins (Figure 1).

**Table 1:** Prediction performance on 50 test sets.

	Sensitivity^1^	Specificity^2^	Accuracy^3^	MCC^4^
Best	0.838	0.926	0.903	0.717
Worst	0.730	0.865	0.838	0.546
Mean	0.758	0.888	0.862	0.605
Standard deviation	0.021	0.017	0.041	0.033

Best and worst performance are selected based on MCC.

1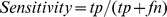.

2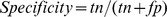.

3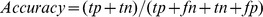.

4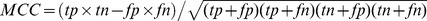.

As shown in previous studies, SVM method based on amino acid composition (termed as SVMaac hereinafter) performs relatively well in prediction of protein subcellular localization [35], [36]. To test the performance of SVMaac in prediction of flagellar proteins, we applied it to the same training and test datasets used in our method. Parameters required for SVM models in training SVMaac were selected using the same method as introduced in “Materials and Methods” section. The prediction performance of SVMaac on 50 test sets was shown in Table S4. We found that the accuracy of SVMaac is acceptable, but the sensitivity is quite low. For all the test sets, less than 60% flagellar proteins can be successfully predicted by SVMaac, which is much lower than the sensitivity of TFPP (83.8%). This is likely due to the intricate sorting system of flagellar proteins. The prediction of flagellar proteins is relatively more complex, and thus amino acid composition alone cannot characterize them well. These results demonstrate that SVM method based on the selected features performs much better than that based on amino acid composition, and thus further confirmed the effectiveness of the selected features.

**Figure 2:**
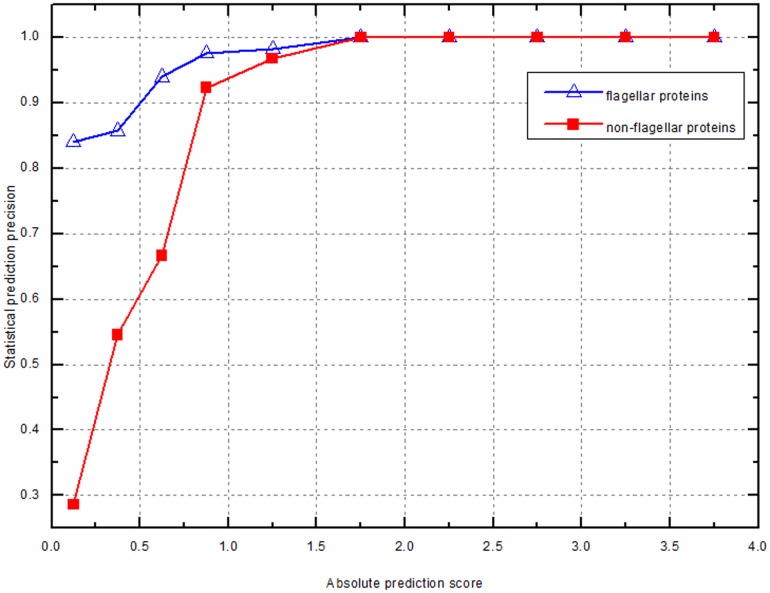
Statistical relationship between the prediction precision and the prediction score. For the purpose of display, the x-axis is the absolute value of prediction score.

When a protein is predicted to be flagellar or non-flagellar protein, it's important to know how confident the prediction is. Thus, we analyzed the relationship between the prediction score and the prediction precision based on the predicted result on the whole dataset. As shown in Figure 2, we can evaluate the reliability of the predicted result as: (1) flagellar protein with high confidence when 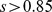, (2) flagellar protein with medium confidence when 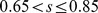, (3) flagellar protein with low confidence when 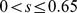, (4) non-flagellar protein with medium confidence when 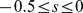, (5) non-flagellar protein with high confidence when 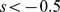. For the whole dataset, TFPP can correctly identify 90.5% flagellar proteins and 96.3% non-flagellar proteins. 88.1% of the predicted flagellar and 93.7% of the predicted non-flagellar proteins have a high confidence value (Table S5).

### TFPP server

To make the software available for users, we developed an online web server for TFPP. It can be freely accessed academically at http:/wukong.tongji.edu.cn/tfpp. TFPP provides an easy-to-use and highly effective platform for identifying flagellar proteins in *T. brucei*. To predict if a protein is targeted to the flagellum or not, the only input of TFPP is the amino acid sequence of the protein. Users can paste the input sequences in the textbox or upload a sequence file in FASTA format. TFPP also provides email notification, it will inform the user when the result is ready. This is very useful especially for a large number of input sequences. As a prediction tool, it is important to tell if the prediction result can be trusted or not. TFPP provides the estimated reliability for each prediction result. Users can selectively use the prediction results with different confidence levels as needed. Moreover, some useful features such as the content of amino acid E, di-peptide EE and exposed amino acids, and surface accessibility information are displayed together with prediction result for each query sequence. To the best of our knowledge, TFPP is the first available computational method for the identification of flagellar proteins in *T. brucei*, and we believe it will greatly benefit research of flagellar biogenesis in trypanosomes.

### Regulation of flagellar proteins during development

As an application, TFPP was used to predict the flagellar proteome in *T. brucei*. The *T. brucei* proteome was downloaded from TriTrypDB Version 4.1 containing 9826 proteins. 8865 proteins were retained after removal of the incomplete entries such as those not beginning with “M”, containing “*” or “X” characters, and less than 50 amino acids in length. Besides the 148 known flagellar proteins, 811 more proteins are assigned to the flagellum with high confidence by TFPP. Moreover, the 8 flagellar and 60 non-flagellar proteins which are removed from our dataset in the redundancy handling process were correctly recognized with high confidence respectively. This suggests that the flagellar proteome of *T. brucei* may contain at least 959 proteins, covering ∼10% of the whole proteome.

As observed in previous studies, the single flagellum of *T. brucei* changes in morphology and function during the life cycle alternating between a tsetse fly and a mammal host [3], [37]. A recent study analyzed the expression profiles of the *T. brucei* proteome at three important life-cycle stages, namely long slender and short stumpy bloodstream forms in the mammalian host and the procyclic form in the midgut of the tsetse fly [38]. Considering uniquely mapped reads, 800 (83.4%) flagellar genes were detected to be expressed in at least one of the three stages. Differentially expressed genes were defined to be those two-fold up- or down-regulated at two stages with 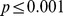 according to the Audic and Claverie test [39]. In total, 363 flagellar protein-encoded genes significantly changed expression levels in at least one of the three stages, accounting for ∼45% of the expressed flagellar proteins (Table 2). As expected, much more flagellar genes changed their expression levels in procyclic form when compared with the other two bloodstream stages. As *T. brucei* lives in from the tsetse fly to the mammal host, the parasite needs more genes to be regulated to adapt to host change for survival. We found that most of these differentially expressed genes were up-regulated in the procyclic form when compared with the long slender and short stumpy bloodstream form. This is not surprising, as we know that the flagellum-mediated migration between the midgut and salivary glands of its tsetse fly vector is essential for the progression of its life cycle [3], [40]. When compared with the short stumpy bloodstream form, we found much more flagellar genes were up-regulated in the long slender and procyclic forms. This may due to the important roles of the single flagellum in cell division as demonstrated by previous studies [5], [40], [41], [42], while both the long slender bloodstream form and the procyclic form are proliferative forms. These results indicate life cycle stage-specific regulation of flagellar functions in *T. brucei*.

**Table 2:** Differential expression of the flagellar proteome in long slender (LS), short stumpy (SS) and procyclic (PC) form *T. brucei*.

	LS/SS	SS/PC	LS/PC	Total
Significantly regulated genes	93	289	256	363^1^
Up-regulated^2^	80	92	125	
Down-regulated^3^	13	197	131	
Genes expressed^4^	782 (LS)	767 (SS)	760 (PC)	800^5^

1 total number of significantly regulated genes.

2 up-regulated in LS compared with SS, SS compared with PC, and LS compared with PC.

3 down-regulated in LS compared with SS, SS compared with PC, and LS compared with PC.

4 genes with uniquely mapped reads in LS, SS and PC form.

5 genes with uniquely mapped reads in at least one of the three life cycle stages.

## Conclusions

The available evidence indicates the multifunctional nature of the single flagellum in *T. brucei*, and suggests a new way to uncover novel drug targets for sleeping sickness. In this study, we developed a novel computational method TFPP to recognize flagellar proteins in *T. brucei*. TFPP effectively identifies a large number of flagellar proteins with high confidence, many of which are reported first time in our study. Expression profiles of the flagellar proteome show that ∼45% flagellar proteins are significantly regulated during life cycle, indicating life cycle stage-specific regulation of flagellar functions in *T. brucei*. We further developed a web server for TFPP with free access. Therefore, TFPP will largely facilitate identification and functional study of flagellar proteins. Moreover, the approach proposed in this study can be extended for application in other flagellated organisms especially trypanosome related species.

## Supporting Information

Table S1 List of positive and negative samples.
(DOC)

Table S2 List of initial features and the element number of each feature.
(DOC)

Table S3 Features selected to build the final classifier model.
(DOC)

Table S4 Prediction performance of SVMaac on 50 test sets.
(DOC)

Table S5 Prediction result of all positive and negative samples by TFPP.
(DOC)
