# Supplementary material for: TFPP: An SVM-Based Tool for Recognizing Flagellar Proteins in Trypanosoma brucei
Source: PLoS One. 2013 Jan 17;8(1):e54032. doi: 10.1371/journal.pone.0054032 (PMC3547966; doi:10.1371/journal.pone.0054032)
Supplement: Table S1 — List of positive and negative samples. (DOC) [file pone.0054032.s001.doc]

**Table S1A. *T. brucei*** Gene IDs of 148 flagellar proteins used as positive samples.

| **No.** | **Gene ID** | **No.** | **Gene ID** | **No.** | **Gene ID** |
| --- | --- | --- | --- | --- | --- |
| 1 | Tb927.3.2310 | 51 | Tb09.160.4520 | 101 | Tb927.8.6660 |
| 2 | Tb11.50.0007 | 52 | Tb927.7.6830 | 102 | Tb927.7.6290 |
| 3 | Tb11.01.5470 | 53 | Tb927.1.2670 | 103 | Tb927.4.5370 |
| 4 | Tb927.8.4010 | 54 | Tb927.10.11080 | 104 | Tb927.5.4480 |
| 5 | Tb11.02.3900 | 55 | Tb11.03.0810 | 105 | Tb11.02.1380 |
| 6 | Tb09.211.2560 | 56 | Tb927.5.2850 | 106 | Tb927.4.1740 |
| 7 | Tb927.7.6950 | 57 | Tb11.01.4400 | 107 | Tb927.10.7960 |
| 8 | Tb09.211.2540 | 58 | Tb927.7.6850 | 108 | Tb927.10.3130 |
| 9 | Tb927.10.11300 | 59 | Tb927.5.3510 | 109 | Tb927.6.1720 |
| 10 | Tb927.3.5510 | 60 | Tb927.8.2630 | 110 | Tb927.3.930 |
| 11 | Tb927.3.4760 | 61 | Tb927.7.3560 | 111 | Tb11.03.0470 |
| 12 | Tb927.2.3020 | 62 | Tb927.6.2720 | 112 | Tb927.6.3920 |
| 13 | Tb927.8.5230 | 63 | Tb927.10.3020 | 113 | Tb927.4.3950 |
| 14 | Tb11.02.5800 | 64 | Tb11.01.6040 | 114 | Tb927.7.5050 |
| 15 | Tb09.160.3960 | 65 | Tb927.3.4290 | 115 | Tb927.4.2140 |
| 16 | Tb11.01.3110 | 66 | Tb11.01.4621 | 116 | Tb927.8.3250 |
| 17 | Tb927.8.6910 | 67 | Tb927.8.780 | 117 | Tb927.10.10360 |
| 18 | Tb11.01.3960 | 68 | Tb927.7.5690 | 118 | Tb927.4.3130 |
| 19 | Tb927.8.3780 | 69 | Tb927.1.2360 | 119 | Tb927.5.2270 |
| 20 | Tb09.211.3470 | 70 | Tb927.7.3450 | 120 | Tb11.01.2670 |
| 21 | Tb11.02.5550 | 71 | Tb927.8.6260 | 121 | Tb11.01.3010 |
| 22 | Tb11.47.0006 | 72 | Tb927.10.15280 | 122 | Tb927.10.8930 |
| 23 | Tb927.7.5650 | 73 | Tb11.02.4320 | 123 | Tb11.01.5910 |
| 24 | Tb927.7.920 | 74 | Tb927.10.13960 | 124 | Tb927.10.14010 |
| 25 | Tb927.5.2950 | 75 | Tb11.01.7750 | 125 | Tb927.10.10280 |
| 26 | Tb11.02.2060 | 76 | Tb11.01.4810 | 126 | Tb11.01.0160 |
| 27 | Tb11.01.6780 | 77 | Tb927.7.3440 | 127 | Tb927.8.4970 |
| 28 | Tb927.10.7690 | 78 | Tb927.3.3690 | 128 | Tb927.5.3630 |
| 29 | Tb11.01.3520 | 79 | Tb11.01.1210 | 129 | Tb11.01.1870 |
| 30 | Tb927.10.6670 | 80 | Tb11.02.1260 | 130 | Tb11.01.5100 |
| 31 | Tb927.3.5310 | 81 | Tb927.7.6970 | 131 | Tb927.1.2330 |
| 32 | Tb927.2.4330 | 82 | Tb11.02.2640 | 132 | Tb927.10.3310 |
| 33 | Tb927.1.4310 | 83 | Tb927.2.5270 | 133 | Tb927.7.4510 |
| 34 | Tb927.4.870 | 84 | Tb927.10.650 | 134 | Tb927.10.5980 |
| 35 | Tb927.3.1990 | 85 | Tb09.211.2450 | 135 | Tb11.02.3200 |
| 36 | Tb927.8.5440 | 86 | Tb927.6.4140 | 136 | Tb11.02.5590 |
| 37 | Tb927.8.4640 | 87 | Tb927.2.4060 | 137 | Tb927.3.3540 |
| 38 | Tb927.5.3970 | 88 | Tb927.5.1730 | 138 | Tb927.6.3150 |
| 39 | Tb927.6.4670 | 89 | Tb11.01.8770 | 139 | Tb927.7.2790 |
| 40 | Tb11.02.0860 | 90 | Tb927.6.5030 | 140 | Tb11.02.1135 |
| 41 | Tb927.10.5350 | 91 | Tb927.5.2530 | 141 | Tb09.160.2070 |
| 42 | Tb11.02.0760 | 92 | Tb927.8.640 | 142 | Tb927.7.820 |
| 43 | Tb09.211.3240 | 93 | Tb927.10.1130 | 143 | Tb11.01.8370 |
| 44 | Tb11.47.0034 | 94 | Tb927.8.4870 | 144 | Tb927.7.1920 |
| 45 | Tb11.01.0390 | 95 | Tb927.3.3770 | 145 | Tb11.02.3390 |
| 46 | Tb927.10.6350 | 96 | Tb927.8.1550 | 146 | Tb927.10.7120 |
| 47 | Tb927.10.3160 | 97 | Tb927.10.9570 | 147 | Tb927.10.2100 |
| 48 | Tb927.4.3330 | 98 | Tb927.3.3750 | 148 | Tb927.8.3790 |
| 49 | Tb927.8.6240 | 99 | Tb927.4.1720 |  |  |
| 50 | Tb927.10.15410 | 100 | Tb927.5.1880 |  |  |

**Table S1B. *T. brucei*** Gene IDs of 592 non-flagellar proteins used as negative samples.

| **No.** | **Gene ID** | **No.** | **Gene ID** | **No.** | **Gene ID** |
| --- | --- | --- | --- | --- | --- |
| 1 | Tb11.01.3860 | 199 | Tb927.10.8890 | 397 | Tb11.02.4810 |
| 2 | Tb927.5.1510 | 200 | Tb927.5.3710 | 398 | Tb927.2.4110 |
| 3 | Tb927.10.3210 | 201 | Tb927.1.1580 | 399 | Tb11.55.0016 |
| 4 | Tb927.10.7380 | 202 | Tb927.7.3510 | 400 | Tb11.52.0006 |
| 5 | Tb11.01.2340 | 203 | Tb927.10.4040 | 401 | Tb927.6.2370 |
| 6 | Tb11.01.8620 | 204 | Tb927.3.5520 | 402 | Tb927.6.2180 |
| 7 | Tb09.160.5240 | 205 | Tb927.7.2570 | 403 | Tb09.211.0690 |
| 8 | Tb927.7.2620 | 206 | Tb927.4.3570 | 404 | Tb927.10.280 |
| 9 | Tb927.2.2510 | 207 | Tb927.8.2070 | 405 | Tb927.10.9720 |
| 10 | Tb927.8.3330 | 208 | Tb927.1.4230 | 406 | Tb11.01.1910 |
| 11 | Tb11.22.0004 | 209 | Tb927.5.450 | 407 | Tb11.02.2070 |
| 12 | Tb927.10.11350 | 210 | Tb09.211.4400 | 408 | Tb927.10.520 |
| 13 | Tb927.3.2230 | 211 | Tb11.01.6800 | 409 | Tb927.8.2540 |
| 14 | Tb927.10.8640 | 212 | Tb927.10.380 | 410 | Tb927.10.7610 |
| 15 | Tb927.10.5670 | 213 | Tb927.6.4930 | 411 | Tb927.10.14630 |
| 16 | Tb927.8.5090 | 214 | Tb927.7.3990 | 412 | Tb927.5.1710 |
| 17 | Tb09.211.2430 | 215 | Tb09.211.4580 | 413 | Tb927.10.2410 |
| 18 | Tb927.10.7090 | 216 | Tb927.3.1790 | 414 | Tb927.5.3640 |
| 19 | Tb11.01.7390 | 217 | Tb927.6.4200 | 415 | Tb11.01.8630 |
| 20 | Tb09.211.4700 | 218 | Tb927.8.1460 | 416 | Tb11.02.4100 |
| 21 | Tb927.4.470 | 219 | Tb927.7.6350 | 417 | Tb11.01.4860 |
| 22 | Tb09.211.0220 | 220 | Tb927.10.4130 | 418 | Tb927.7.2630 |
| 23 | Tb927.8.4440 | 221 | Tb927.10.9820 | 419 | Tb09.160.5250 |
| 24 | Tb11.02.4000 | 222 | Tb927.8.1890 | 420 | Tb927.10.5500 |
| 25 | Tb927.8.6060 | 223 | Tb927.10.7500 | 421 | Tb927.8.3320 |
| 26 | Tb927.10.5400 | 224 | Tb11.02.0770 | 422 | Tb11.46.0001 |
| 27 | Tb927.10.3690 | 225 | Tb927.10.3120 | 423 | Tb927.10.16090 |
| 28 | Tb927.10.13600 | 226 | Tb927.8.2080 | 424 | Tb927.8.5880 |
| 29 | Tb927.10.8030 | 227 | Tb11.01.3020 | 425 | Tb927.6.4080 |
| 30 | Tb11.01.1160 | 228 | Tb927.7.3050 | 426 | Tb11.02.5530 |
| 31 | Tb927.5.3220 | 229 | Tb11.02.5160 | 427 | Tb927.8.8150 |
| 32 | Tb11.02.0530 | 230 | Tb11.03.0580 | 428 | Tb927.1.4500 |
| 33 | Tb11.01.7810 | 231 | Tb927.4.4380 | 429 | Tb11.01.1740 |
| 34 | Tb09.160.4750 | 232 | Tb11.02.4800 | 430 | Tb927.5.3360 |
| 35 | Tb927.3.2180 | 233 | Tb11.01.1440 | 431 | Tb11.02.3670 |
| 36 | Tb927.6.2010 | 234 | Tb927.2.2090 | 432 | Tb927.10.13290 |
| 37 | Tb927.2.4400 | 235 | Tb11.55.0009 | 433 | Tb09.244.2590 |
| 38 | Tb927.10.2230 | 236 | Tb927.10.2880 | 434 | Tb11.03.0030 |
| 39 | Tb11.02.0490 | 237 | Tb927.10.10830 | 435 | Tb09.211.0230 |
| 40 | Tb11.01.5390 | 238 | Tb927.5.1790 | 436 | Tb09.160.4130 |
| 41 | Tb11.01.8470 | 239 | Tb927.10.5220 | 437 | Tb927.10.16170 |
| 42 | Tb927.8.2740 | 240 | Tb11.02.1930 | 438 | Tb11.02.2280 |
| 43 | Tb927.6.3840 | 241 | Tb927.10.14860 | 439 | Tb927.4.760 |
| 44 | Tb927.8.3690 | 242 | Tb11.03.0620 | 440 | Tb927.5.2780 |
| 45 | Tb927.10.14170 | 243 | Tb927.6.2790 | 441 | Tb927.8.680 |
| 46 | Tb927.7.2760 | 244 | Tb11.02.2740 | 442 | Tb927.5.3090 |
| 47 | Tb927.7.3960 | 245 | Tb927.7.4140 | 443 | Tb927.2.5980 |
| 48 | Tb927.8.6970 | 246 | Tb927.4.4620 | 444 | Tb11.02.3130 |
| 49 | Tb927.2.4380 | 247 | Tb09.160.4560 | 445 | Tb927.7.1320 |
| 50 | Tb927.10.12540 | 248 | Tb927.8.5200 | 446 | Tb11.01.2170 |
| 51 | Tb927.6.4540 | 249 | Tb927.7.6990 | 447 | Tb927.5.3560 |
| 52 | Tb927.6.2080 | 250 | Tb927.8.2550 | 448 | Tb927.4.2450 |
| 53 | Tb927.8.2380 | 251 | Tb09.211.1380 | 449 | Tb927.10.12000 |
| 54 | Tb927.8.630 | 252 | Tb927.1.3450 | 450 | Tb09.211.2730 |
| 55 | Tb927.8.6080 | 253 | Tb927.10.5320 | 451 | Tb927.8.1240 |
| 56 | Tb927.4.1860 | 254 | Tb11.01.3500 | 452 | Tb927.10.1680 |
| 57 | Tb11.02.2710 | 255 | Tb927.10.15710 | 453 | Tb11.01.6260 |
| 58 | Tb09.244.2790 | 256 | Tb927.10.2770 | 454 | Tb927.8.3060 |
| 59 | Tb927.5.2070 | 257 | Tb11.01.2000 | 455 | Tb927.7.3470 |
| 60 | Tb11.02.4540 | 258 | Tb927.10.3370 | 456 | Tb927.5.2580 |
| 61 | Tb11.02.5660 | 259 | Tb927.2.6200 | 457 | Tb927.4.3690 |
| 62 | Tb09.211.1320 | 260 | Tb11.01.4870 | 458 | Tb927.10.10610 |
| 63 | Tb927.6.3930 | 261 | Tb927.2.2470 | 459 | Tb927.10.5120 |
| 64 | Tb927.4.1810 | 262 | Tb927.4.310 | 460 | Tb927.4.5010 |
| 65 | Tb11.01.5710 | 263 | Tb09.160.5260 | 461 | Tb927.5.1210 |
| 66 | Tb927.1.1690 | 264 | Tb927.10.1870 | 462 | Tb927.4.3450 |
| 67 | Tb927.6.2420 | 265 | Tb927.8.3310 | 463 | Tb927.10.9440 |
| 68 | Tb11.01.6880 | 266 | Tb927.3.820 | 464 | Tb927.8.2760 |
| 69 | Tb927.10.8210 | 267 | Tb09.354.0090 | 465 | Tb927.4.4160 |
| 70 | Tb11.47.0004 | 268 | Tb927.7.1550 | 466 | Tb11.01.7110 |
| 71 | Tb927.2.2940 | 269 | Tb927.8.2020 | 467 | Tb927.5.4040 |
| 72 | Tb927.5.890 | 270 | Tb927.2.3320 | 468 | Tb927.8.5280 |
| 73 | Tb927.10.14280 | 271 | Tb927.10.13300 | 469 | Tb927.3.770 |
| 74 | Tb11.01.7400 | 272 | Tb927.8.8120 | 470 | Tb927.1.1160 |
| 75 | Tb927.7.2820 | 273 | Tb927.10.200 | 471 | Tb927.2.6070 |
| 76 | Tb927.10.11260 | 274 | Tb11.01.5960 | 472 | Tb927.4.1070 |
| 77 | Tb11.01.1930 | 275 | Tb927.10.7330 | 473 | Tb927.10.12050 |
| 78 | Tb927.3.1710 | 276 | Tb927.3.2260 | 474 | Tb927.8.4040 |
| 79 | Tb09.160.4570 | 277 | Tb927.3.1000 | 475 | Tb11.01.4701 |
| 80 | Tb927.4.4610 | 278 | Tb927.2.2970 | 476 | Tb927.8.8280 |
| 81 | Tb11.03.0180 | 279 | Tb927.8.2430 | 477 | Tb09.160.4590 |
| 82 | Tb927.7.2990 | 280 | Tb927.5.3010 | 478 | Tb927.7.1070 |
| 83 | Tb09.211.4740 | 281 | Tb11.02.0350 | 479 | Tb927.3.3220 |
| 84 | Tb927.7.3910 | 282 | Tb927.5.3870 | 480 | Tb11.01.2680 |
| 85 | Tb927.8.7720 | 283 | Tb927.4.4870 | 481 | Tb927.1.3830 |
| 86 | Tb927.10.5330 | 284 | Tb927.10.3870 | 482 | Tb927.10.12700 |
| 87 | Tb927.7.3620 | 285 | Tb11.02.1110 | 483 | Tb927.10.2840 |
| 88 | Tb11.03.0475 | 286 | Tb927.8.2130 | 484 | Tb09.211.3800 |
| 89 | Tb11.02.2390 | 287 | Tb09.160.0680 | 485 | Tb927.10.15010 |
| 90 | Tb927.10.5440 | 288 | Tb927.10.13740 | 486 | Tb11.02.5770 |
| 91 | Tb09.160.0780 | 289 | Tb927.10.1160 | 487 | Tb11.01.3675 |
| 92 | Tb927.8.3300 | 290 | Tb11.01.1840 | 488 | Tb11.01.8200 |
| 93 | Tb927.7.4710 | 291 | Tb927.10.340 | 489 | Tb09.160.1820 |
| 94 | Tb11.01.6620 | 292 | Tb11.02.2480 | 490 | Tb11.02.5670 |
| 95 | Tb09.160.2970 | 293 | Tb927.8.2530 | 491 | Tb09.244.2620 |
| 96 | Tb927.2.4590 | 294 | Tb927.8.4250 | 492 | Tb927.10.6640 |
| 97 | Tb927.8.1590 | 295 | Tb927.10.13150 | 493 | Tb927.2.6280 |
| 98 | Tb927.10.2220 | 296 | Tb927.3.5240 | 494 | Tb927.8.3750 |
| 99 | Tb927.5.1300 | 297 | Tb927.7.3940 | 495 | Tb09.211.4550 |
| 100 | Tb927.7.3950 | 298 | Tb927.10.3570 | 496 | Tb09.160.3800 |
| 101 | Tb11.39.0006 | 299 | Tb927.5.4120 | 497 | Tb927.6.1250 |
| 102 | Tb11.39.0007 | 300 | Tb927.6.720 | 498 | Tb11.47.0012 |
| 103 | Tb927.5.900 | 301 | Tb11.03.0870 | 499 | Tb11.55.0026 |
| 104 | Tb927.10.470 | 302 | Tb927.10.16120 | 500 | Tb927.8.7400 |
| 105 | Tb927.10.600 | 303 | Tb11.02.0730 | 501 | Tb11.47.0017 |
| 106 | Tb927.7.7080 | 304 | Tb927.10.14690 | 502 | Tb11.0400 |
| 107 | Tb927.6.3600 | 305 | Tb927.10.14750 | 503 | Tb11.46.0006 |
| 108 | Tb927.3.1590 | 306 | Tb927.7.5170 | 504 | Tb927.10.13820 |
| 109 | Tb11.02.1106 | 307 | Tb927.7.840 | 505 | Tb927.1.4100 |
| 110 | Tb09.211.0560 | 308 | Tb927.5.3410 | 506 | Tb09.244.2840 |
| 111 | Tb927.5.3980 | 309 | Tb927.10.4590 | 507 | Tb927.8.1060 |
| 112 | Tb927.5.1060 | 310 | Tb11.01.5060 | 508 | Tb927.4.4600 |
| 113 | Tb927.7.1730 | 311 | Tb927.10.1620 | 509 | Tb927.5.3320 |
| 114 | Tb927.8.580 | 312 | Tb11.01.5180 | 510 | Tb927.6.930 |
| 115 | Tb11.01.7140 | 313 | Tb927.10.4110 | 511 | Tb927.8.7170 |
| 116 | Tb09.211.3000 | 314 | Tb927.10.11900 | 512 | Tb11.01.0480 |
| 117 | Tb927.3.3660 | 315 | Tb11.02.5280 | 513 | Tb927.10.9120 |
| 118 | Tb927.8.5640 | 316 | Tb927.6.590 | 514 | Tb927.6.4500 |
| 119 | Tb927.10.4770 | 317 | Tb927.4.4830 | 515 | Tb927.3.5610 |
| 120 | Tb11.02.3530 | 318 | Tb11.01.7510 | 516 | Tb927.5.1550 |
| 121 | Tb11.02.0440 | 319 | Tb927.2.5930 | 517 | Tb927.10.3250 |
| 122 | Tb927.7.1300 | 320 | Tb927.10.14820 | 518 | Tb927.4.720 |
| 123 | Tb09.160.2020 | 321 | Tb927.6.4560 | 519 | Tb927.8.6150 |
| 124 | Tb09.160.3820 | 322 | Tb927.7.6360 | 520 | Tb927.8.7530 |
| 125 | Tb927.3.1840 | 323 | Tb927.10.10420 | 521 | Tb09.160.5280 |
| 126 | Tb11.02.0620 | 324 | Tb11.01.7300 | 522 | Tb927.10.8040 |
| 127 | Tb927.4.2720 | 325 | Tb927.8.5710 | 523 | Tb927.3.1890 |
| 128 | Tb11.02.2300 | 326 | Tb927.8.5560 | 524 | Tb09.211.1600 |
| 129 | Tb927.10.6300 | 327 | Tb09.211.1220 | 525 | Tb11.01.1600 |
| 130 | Tb927.7.7010 | 328 | Tb927.5.3040 | 526 | Tb927.3.1410 |
| 131 | Tb927.10.8390 | 329 | Tb09.211.4540 | 527 | Tb927.8.4550 |
| 132 | Tb927.8.1860 | 330 | Tb927.2.4210 | 528 | Tb927.7.1040 |
| 133 | Tb927.10.2440 | 331 | Tb927.6.3500 | 529 | Tb11.01.7480 |
| 134 | Tb927.8.8180 | 332 | Tb09.211.3200 | 530 | Tb11.02.4480 |
| 135 | Tb11.01.1475 | 333 | Tb927.10.8230 | 531 | Tb927.3.3270 |
| 136 | Tb927.6.2170 | 334 | Tb09.211.1830 | 532 | Tb927.10.13320 |
| 137 | Tb927.7.1740 | 335 | Tb927.7.4180 | 533 | Tb927.5.3350 |
| 138 | Tb11.01.1470 | 336 | Tb11.47.0024 | 534 | Tb927.7.6800 |
| 139 | Tb927.8.6390 | 337 | Tb11.47.0022 | 535 | Tb927.5.1520 |
| 140 | Tb09.160.4380 | 338 | Tb11.01.2280 | 536 | Tb09.160.0760 |
| 141 | Tb11.02.3800 | 339 | Tb927.8.6960 | 537 | Tb11.01.7290 |
| 142 | Tb09.211.0180 | 340 | Tb927.2.1700 | 538 | Tb927.3.1940 |
| 143 | Tb11.01.5570 | 341 | Tb09.160.3110 | 539 | Tb927.6.4040 |
| 144 | Tb09.244.2730 | 342 | Tb927.3.750 | 540 | Tb927.10.1270 |
| 145 | Tb927.10.5360 | 343 | Tb11.02.5420 | 541 | Tb927.7.3590 |
| 146 | Tb927.8.5860 | 344 | Tb927.8.4890 | 542 | Tb11.01.4702 |
| 147 | Tb927.3.1690 | 345 | Tb09.160.2250 | 543 | Tb11.02.3210 |
| 148 | Tb927.8.1420 | 346 | Tb11.01.1810 | 544 | Tb09.211.4850 |
| 149 | Tb927.7.7330 | 347 | Tb927.8.8170 | 545 | Tb11.02.2250 |
| 150 | Tb11.02.0130 | 348 | Tb11.01.5480 | 546 | Tb927.10.6200 |
| 151 | Tb09.160.4200 | 349 | Tb09.211.1900 | 547 | Tb927.10.2010 |
| 152 | Tb927.6.1410 | 350 | Tb11.01.1690 | 548 | Tb11.02.4890 |
| 153 | Tb11.01.3300 | 351 | Tb927.3.590 | 549 | Tb11.02.1480 |
| 154 | Tb09.v1.0420 | 352 | Tb927.6.1440 | 550 | Tb927.10.10130 |
| 155 | Tb11.01.3710 | 353 | Tb927.1.4490 | 551 | Tb927.1.2990 |
| 156 | Tb927.4.4910 | 354 | Tb927.7.2340 | 552 | Tb11.02.2460 |
| 157 | Tb927.1.5300 | 355 | Tb11.02.5390 | 553 | Tb927.6.4130 |
| 158 | Tb09.211.3540 | 356 | Tb927.8.5120 | 554 | Tb927.2.3610 |
| 159 | Tb927.10.8320 | 357 | Tb11.02.2960 | 555 | Tb927.7.7440 |
| 160 | Tb927.6.4580 | 358 | Tb09.160.2810 | 556 | Tb11.01.4740 |
| 161 | Tb927.10.6780 | 359 | Tb09.211.2580 | 557 | Tb927.4.1330 |
| 162 | Tb11.02.1440 | 360 | Tb927.2.6000 | 558 | Tb09.211.3650 |
| 163 | Tb927.8.5690 | 361 | Tb927.10.3040 | 559 | Tb927.10.8970 |
| 164 | Tb11.01.1650 | 362 | Tb09.211.1770 | 560 | Tb927.10.5050 |
| 165 | Tb11.02.3180 | 363 | Tb11.01.6600 | 561 | Tb09.160.3520 |
| 166 | Tb927.8.3170 | 364 | Tb927.8.4860 | 562 | Tb11.03.0950 |
| 167 | Tb927.2.5870 | 365 | Tb927.10.4880 | 563 | Tb09.160.4300 |
| 168 | Tb927.3.970 | 366 | Tb11.01.0640 | 564 | Tb11.02.0610 |
| 169 | Tb927.3.2980 | 367 | Tb11.01.7090 | 565 | Tb927.10.8430 |
| 170 | Tb927.10.11160 | 368 | Tb927.6.3640 | 566 | Tb09.211.0290 |
| 171 | Tb11.02.2700 | 369 | Tb927.4.2880 | 567 | Tb09.160.4090 |
| 172 | Tb927.8.1490 | 370 | Tb927.2.3030 | 568 | Tb927.8.1870 |
| 173 | Tb927.5.3300 | 371 | Tb927.10.660 | 569 | Tb09.211.4510 |
| 174 | Tb11.02.4170 | 372 | Tb11.03.0260 | 570 | Tb11.01.2560 |
| 175 | Tb927.4.440 | 373 | Tb927.2.6220 | 571 | Tb927.2.4890 |
| 176 | Tb927.10.7060 | 374 | Tb09.211.0540 | 572 | Tb11.01.5040 |
| 177 | Tb927.1.730 | 375 | Tb927.10.11820 | 573 | Tb09.160.2770 |
| 178 | Tb11.01.0170 | 376 | Tb927.4.4300 | 574 | Tb11.02.3810 |
| 179 | Tb927.8.6030 | 377 | Tb927.7.800 | 575 | Tb927.2.3800 |
| 180 | Tb927.7.5260 | 378 | Tb927.10.13620 | 576 | Tb927.10.1390 |
| 181 | Tb927.1.3030 | 379 | Tb927.5.360 | 577 | Tb927.8.6840 |
| 182 | Tb927.7.3460 | 380 | Tb927.4.1500 | 578 | Tb09.244.2720 |
| 183 | Tb927.3.5630 | 381 | Tb11.01.1130 | 579 | Tb927.10.3260 |
| 184 | Tb927.3.3090 | 382 | Tb927.10.13120 | 580 | Tb927.8.1430 |
| 185 | Tb927.1.4830 | 383 | Tb927.3.5050 | 581 | Tb11.01.1900 |
| 186 | Tb927.8.2100 | 384 | Tb927.10.11390 | 582 | Tb09.211.0680 |
| 187 | Tb927.7.2370 | 385 | Tb927.5.3110 | 583 | Tb927.10.7410 |
| 188 | Tb927.10.13630 | 386 | Tb927.10.6850 | 584 | Tb927.3.2880 |
| 189 | Tb927.2.1860 | 387 | Tb927.6.4210 | 585 | Tb927.10.12630 |
| 190 | Tb09.211.2020 | 388 | Tb09.211.1510 | 586 | Tb927.7.3240 |
| 191 | Tb927.7.7410 | 389 | Tb09.160.4310 | 587 | Tb927.10.10400 |
| 192 | Tb927.8.3290 | 390 | Tb927.10.8830 | 588 | Tb927.8.3110 |
| 193 | Tb11.01.1780 | 391 | Tb927.10.11050 | 589 | Tb11.02.0290 |
| 194 | Tb927.10.3580 | 392 | Tb09.244.2670 | 590 | Tb927.1.1200 |
| 195 | Tb927.8.760 | 393 | Tb927.8.1880 | 591 | Tb11.01.5590 |
| 196 | Tb927.10.12940 | 394 | Tb11.01.2420 | 592 | Tb927.10.180 |
| 197 | Tb927.6.940 | 395 | Tb11.01.8090 |  |  |
| 198 | Tb927.10.4000 | 396 | Tb927.3.860 |  |  |
