# Supplementary material for: TFPP: An SVM-Based Tool for Recognizing Flagellar Proteins in Trypanosoma brucei
Source: PLoS One. 2013 Jan 17;8(1):e54032. doi: 10.1371/journal.pone.0054032 (PMC3547966; doi:10.1371/journal.pone.0054032)
Supplement: Table S2 — List of initial features and the element number of each feature. (DOC) [file pone.0054032.s002.doc]

**Table S2. List of initial features and the element number of each feature.**

| **Type of properties** | **Features (dimension)** | **Sources [reference]** |
| --- | --- | --- |
| Basic sequence  attributes | Sequence length (1)  Amino acid composition (20)  Di-peptide composition (400) | Locally calculated |
| Physicochemical and biochemical  properties | Amino acid propensities (544) | Locally calculated based on the amino acid indices obtained from AAindex |
|  | Extinction coefficient (4)  Instability index (1)  Aliphatic index (1)  Grand average of hydropathicity (1)  Isoelectric point (1)  Molecular weight (1) | ProtParam |
| Structural properties | Solvent accessibility (4)  Secondary structural content (3) | NetSurfP 1.1 |
|  | Unfoldability (1)  Disordered regions (3)  Global charge (1)  Hydrophobicity (1) | FoldIndex |
| Signal peptide and  transmembrane topology | Signal peptide (2) | SignalP 4.0 |
|  | Transmembrane domains  (alpha-helix and beta-barrel) (3) | TMHMM 2.0  TMB-Hunt |
| Post-translational modifications | Phosphorylation (3) | NetPhos 2.0 |
|  | Acetylation (1) | NetAcet 1.0 |
|  | Palmitoylation (4) | CSS-Palm 3.0 |

1. Kawashima S, Pokarowski P, Pokarowska M, Kolinski A, Katayama T, et al. (2008) AAindex: amino acid index database, progress report 2008. Nucleic Acids Res 36: D202-205.

2. Gasteiger E, Hoogland C, Gattiker A, Duvaud S, Wilkins MR, et al. (2005) Protein identification and analysis tools on the ExPASy server. (In) John M. Walker (ed). The proteomics protocols handbook Humana Press: pp. 571-607.

3. Petersen B, Petersen TN, Andersen P, Nielsen M, Lundegaard C (2009) A generic method for assignment of reliability scores applied to solvent accessibility predictions. BMC Struct Biol 9: 51.

4. Prilusky J, Felder CE, Zeev-Ben-Mordehai T, Rydberg EH, Man O, et al. (2005) FoldIndex: a simple tool to predict whether a given protein sequence is intrinsically unfolded. Bioinformatics 21: 3435-3438.

5. Petersen TN, Brunak S, von Heijne G, Nielsen H (2011) SignalP 4.0: discriminating signal peptides from transmembrane regions. Nat Methods 8: 785-786.

6. Krogh A, Larsson B, von Heijne G, Sonnhammer EL (2001) Predicting transmembrane protein topology with a hidden Markov model: application to complete genomes. J Mol Biol 305: 567-580.

7. Garrow AG, Agnew A, Westhead DR (2005) TMB-Hunt: a web server to screen sequence sets for transmembrane beta-barrel proteins. Nucleic Acids Res 33: W188-192.

8. Blom N, Gammeltoft S, Brunak S (1999) Sequence and structure-based prediction of eukaryotic protein phosphorylation sites. J Mol Biol 294: 1351-1362.

9. Kiemer L, Bendtsen JD, Blom N (2005) NetAcet: prediction of N-terminal acetylation sites. Bioinformatics 21: 1269-1270.

10. Ren J, Wen L, Gao X, Jin C, Xue Y, et al. (2008) CSS-Palm 2.0: an updated software for palmitoylation sites prediction. Protein Eng Des Sel 21: 639-644.
