# Supplementary material for: TFPP: An SVM-Based Tool for Recognizing Flagellar Proteins in Trypanosoma brucei
Source: PLoS One. 2013 Jan 17;8(1):e54032. doi: 10.1371/journal.pone.0054032 (PMC3547966; doi:10.1371/journal.pone.0054032)
Supplement: Table S3 — Features selected to build the final classifier model. (DOC) [file pone.0054032.s003.doc]

**Table S3. Features selected by our feature selection procedure and used to build the final classifier model.** First column, feature description. Except those noted below the table, all of the others are accession numbers of amino acid indices from AAindex. Second and third columns, the mean and standard deviation of flagellar and non-flagellar proteins respectively. Fourth column, F-score. Fifth column, p-value from ANOVA. These features are listed in order of F-score.

| **Feature** | 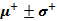 | 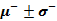 | **F-score** | **p-value** |
| --- | --- | --- | --- | --- |
| E ^1^ | 0.090±0.030 | 0.064±0.021 | 0.207 | 4.73E-32 |
| WIMW960101 | 3.996±0.086 | 4.083±0.075 | 0.203 | 1.64E-31 |
| RICJ880106 | 1.071±0.089 | 0.990±0.067 | 0.202 | 2.17E-31 |
| MEEJ800101 | 0.680±0.792 | 1.460±0.692 | 0.192 | 5.42E-30 |
| Exposed ^2^ | 0.626±0.124 | 0.518±0.096 | 0.179 | 3.35E-28 |
| Buried ^3^ | 0.374±0.124 | 0.482±0.096 | 0.179 | 3.35E-28 |
| RSA ^4^ | 0.345±0.059 | 0.300±0.038 | 0.173 | 1.89E-27 |
| FAUJ880110 | 1.431±0.158 | 1.276±0.148 | 0.169 | 6.70E-27 |
| PARS000102 | 0.363±0.008 | 0.356±0.006 | 0.163 | 5.35E-26 |
| ROBB760102 | -0.070±0.460 | -0.485±0.415 | 0.154 | 9.32E-25 |
| ASA ^5^ | 55.327±10.396 | 48.269±6.266 | 0.151 | 2.42E-24 |
| GEIM800106 | 0.971±0.023 | 0.993±0.024 | 0.138 | 1.84E-22 |
| KLEP840101 | -0.023±0.035 | 0.017±0.045 | 0.138 | 1.60E-22 |
| SNEP660103 | -0.053±0.014 | -0.036±0.019 | 0.135 | 4.29E-22 |
| OOBM850102 | 1.318±0.069 | 1.235±0.098 | 0.128 | 4.27E-21 |
| FAUJ880108 | 0.037±0.003 | 0.034±0.004 | 0.124 | 1.68E-20 |
| HOPA770101 | 2.321±0.211 | 2.147±0.200 | 0.118 | 1.24E-19 |
| FUKS010102 | 6.048±0.348 | 5.768±0.325 | 0.115 | 3.54E-19 |
| LAWE840101 | 0.119±0.072 | 0.176±0.067 | 0.111 | 1.45E-18 |
| MEEJ800102 | 1.311±0.630 | 1.833±0.634 | 0.109 | 2.26E-18 |
| MAXF760102 | 0.980±0.025 | 0.997±0.020 | 0.106 | 6.24E-18 |
| SNEP660102 | 0.007±0.029 | 0.028±0.026 | 0.103 | 1.93E-17 |
| PALJ810108 | 1.059±0.043 | 1.032±0.033 | 0.101 | 3.17E-17 |
| QIAN880118 | -0.018±0.030 | 0.007±0.032 | 0.101 | 3.87E-17 |
| BLAS910101 | 0.483±0.034 | 0.510±0.034 | 0.101 | 3.33E-17 |
| VINM940103 | 1.006±0.003 | 1.003±0.003 | 0.1 | 5.67E-17 |
| GOLD730101 | 1.093±0.089 | 1.167±0.095 | 0.098 | 8.63E-17 |
| OOBM770103 | -0.526±0.013 | -0.536±0.014 | 0.097 | 1.50E-16 |
| RICJ880105 | 1.023±0.054 | 0.982±0.052 | 0.097 | 1.33E-16 |
| EE ^6^ | 0.012±0.010 | 0.006±0.006 | 0.097 | 1.47E-16 |
| FUKS010101 | 6.209±0.461 | 5.884±0.412 | 0.095 | 2.73E-16 |
| VINM940101 | 1.004±0.006 | 0.999±0.006 | 0.094 | 4.04E-16 |
| CASG920101 | -0.239±0.091 | -0.167±0.095 | 0.094 | 4.48E-16 |
| PARJ860101 | 1.822±0.508 | 1.403±0.569 | 0.091 | 1.30E-15 |
| ROSM880105 | -0.673±0.197 | -0.517±0.209 | 0.091 | 1.00E-15 |
| HOPT810101 | 0.215±0.202 | 0.061±0.207 | 0.09 | 1.35E-15 |
| KOEP990102 | 0.288±0.066 | 0.238±0.067 | 0.09 | 1.35E-15 |

^1^ Composition of Glutamic acid

^2^ Composition of the exposed residues

^3^ Composition of the buried residues

^4^ Relative surface accessibility

^5^ Absolute surface accessibility

^6^ Composition of dipeptide EE
