# Supplementary material for: TFPP: An SVM-Based Tool for Recognizing Flagellar Proteins in Trypanosoma brucei
Source: PLoS One. 2013 Jan 17;8(1):e54032. doi: 10.1371/journal.pone.0054032 (PMC3547966; doi:10.1371/journal.pone.0054032)
Supplement: Table S4 — Prediction performance of SVMaac on 50 test sets. (DOC) [file pone.0054032.s004.doc]

**Table S4. Prediction performance of SVMaac on 50 test sets.** Best and worst performances were selected based on MCC.

|  | Sensitivity | Specificity | Accuracy | MCC |
| --- | --- | --- | --- | --- |
| Best | 0.541 | 0.939 | 0.859 | 0.528 |
| Worst | 0.459 | 0.872 | 0.789 | 0.335 |
| Mean | 0.513 | 0.897 | 0.820 | 0.427 |
| Standard deviation | 0.080 | 0.038 | 0.021 | 0.050 |
