# Supplementary material for: TFPP: An SVM-Based Tool for Recognizing Flagellar Proteins in Trypanosoma brucei
Source: PLoS One. 2013 Jan 17;8(1):e54032. doi: 10.1371/journal.pone.0054032 (PMC3547966; doi:10.1371/journal.pone.0054032)
Supplement: Table S5 — Prediction result of all positive and negative samples by TFPP. (DOC) [file pone.0054032.s005.doc]

**Table S5. Prediction result of the whole positive and negative samples by TFPP.**

|  | **High-conf(%^a^)** | **Medium-conf(%^a^)** | **Low-conf(%^a^)** | **Total(%^b^)** |
| --- | --- | --- | --- | --- |
| **Flagellar protein** | 118 (88.1%) | 3 (2.2%) | 13 (9.7%) | 134 (90.5%) |
| **Non-flagellar protein** | 534 (93.7%) | 36 (6.3%) | 0 | 570 (96.3%) |
| **Total** | 652 (92.6%) | 39 (5.5%) | 13 (1.8%) | 704 (95.1%) |

^a^ percentage of predicted proteins regardless of the confidence level

^b^ percentage of total proteins for each category
